# Exploring a Software Framework for Posture Tracking and Haptic Feedback Control: A Virtual Reality-Based Approach for Upper Limb Rehabilitation on the Oculus Quest 2

**DOI:** 10.3390/s25020340

**Published:** 2025-01-09

**Authors:** Joaquin Dillen, Antonio H. J. Moreira, João L. Vilaça

**Affiliations:** 1 2Ai, School of Technology, IPCA, 4750-810 Barcelos, Portugal; amoreira@ipca.pt (A.H.J.M.); jvilaca@ipca.pt (J.L.V.); 2 LASI—Associate Laboratory of Intelligent Systems, 4800-058 Guimarães, Portugal

**Keywords:** virtual reality, Oculus Quest 2, upper limb rehabilitation, physiotherapy

## Abstract

Virtual reality (VR) has gained significant attention in various fields including healthcare and industrial applications. Within healthcare, an interesting application of VR can be found in the field of physiotherapy. The conventional methodology for rehabilitating upper limb lesions is often perceived as tedious and uncomfortable. The manual nature of the process, performed by physicians, leaves patients in an environment lacking motivation and engagement. This presents an opportunity for implementing VR as a tool to enhance the rehabilitation process and improve the quality, efficiency, and evolution of recovery. However, physiotherapy often lacks relevant data to track the recovery process effectively, further compounding concerns about its efficacy. To address this, we propose the development of a posture control system using the Oculus Quest 2, a VR device. Our primary objective was to validate the performance aspects of this device and assess its potential as a rehabilitation tool, providing valuable support to healthcare professionals. Through a series of tests, we evaluated the effectiveness of our VR solution by integrating it into specific therapeutic exercises. This approach enhances patient involvement by offering real-time feedback on exercise execution and providing clear instructions for posture correction. The results demonstrate a notable impact on exercise performance, highlighting the feasibility of developing physiotherapeutically adapted solutions utilizing VR technology. By leveraging the Oculus Quest 2 system and the proposed framework, our research contributes to the advancement of VR-based rehabilitation practices. The findings offer valuable insights into the potential benefits of integrating immersive technologies into the field of physiotherapy, empowering healthcare professionals in their treatment approaches.

## 1. Introduction

Chronic pain (CP) is a major public health issue with important individual consequences and high socio-economic burden [1,2]. Chronic pain affects an estimated 100 million people across Europe, and 50 million Americans live with chronic pain [3], showing that it has reached epidemic proportions worldwide, and its economic costs have been shown to be exceedingly high. Estimates suggest that chronic pain costs the U.S. at least USD 560–635 billion a year in direct medical costs and lost productivity [4].

Shoulder pain plays a significant role in the prevalence of chronic pain conditions originating from the musculoskeletal system. It is recognized as the third most prevalent pain condition encountered in primary healthcare settings. Additionally, it can be associated with various other impairments. It affects both genders [5,6,7] and can be observed across various age groups, particularly in those of working age and increasingly in younger individuals due to occupations involving computer use. The impact of shoulder pain extends to work and domestic activities as well as sports and leisure pursuits [8,9]. The recovery process for shoulder pain can be time-consuming, leading to a high number of persistent and recurrent cases [10,11]. Studies have noted the persistence of shoulder pain even after 12 [10,11,12] or 24 [10,13] months from its onset, which may contribute to the overall prevalence. In fact, shoulder complaints often result in unfavorable outcomes, with only approximately 50% of new episodes of shoulder complaints showing complete recovery within six months of medical intervention [14]. However, this proportion increases to 60% after one year [14].

Exercise-based physiotherapy is widely recognized as the primary treatment for shoulder pain [10,15]. It also plays a crucial role in the rehabilitation process following shoulder surgery [16,17]. Adhering to the prescribed physiotherapy regimen is essential for successful recovery in both conservative [18] and surgical cases [14,16,19]. This includes attending clinical appointments, actively participating in supervised rehabilitation activities guided by a physiotherapist, performing prescribed exercises and activities at home, avoiding potentially harmful actions, and utilizing protective or therapeutic devices [20].

However, adherence to physiotherapy can often be poor, particularly in home settings and among worker populations [21,22]. Furthermore, implementing exercise-based therapy at home poses challenges due to difficulties in supervision. These challenges are particularly relevant in shoulder pain conditions, as they are associated with changes in scapular kinematics and impaired muscular activity. One crucial aspect of the physiotherapist’s role in shoulder pain rehabilitation is to oversee the process of kinematic re-training. This involves ensuring the appropriate recruitment of muscles and the development and maintenance of correct motion patterns during exercise [23]. Achieving this level of supervision and guidance can be particularly challenging in a home-based exercise program.

These limitations contribute to the limited evidence [10] or the controversial findings [9,24,25] regarding the efficacy of exercise-based physiotherapy for shoulder pain.

These findings put the exercise based in need of an additional data driven approach, which becomes more important as we need to constantly measure the improvement of patients along the rehabilitation process. To enhance the methodology in which rehabilitation is approached nowadays, new systems should be developed to provide solid metrics about the performance, execution, and characterization of rehabilitation practices.

In the intention to capture information to present meaningful insights in the form of a report, detailed data needs to be extracted from the realization of the exercises. Some studies point toward the possibility of extracting more detailed information [26,27] through a 3D-reconstructed model of the subject, which is already a goal for virtual reality and could possibly be implemented for the medical purposes described in this paper. Research shows that it is possible to approach an evaluation of the spinal curvature in a non-invasive and radiation-free way. Merging this information allows one to create more personalized programs and study the evolution by implementing real-life scenarios within virtual reality that require appropriate posture to help patients develop good posture habits and prevent further spinal curvature issues.

For more critical medical cases, the described tool could be combined with peripheral hardware [28,29] to extract redundant and robust information. This approach has led to several studies with the implementation of IMU sensors, and the accuracy and robustness of the system will provide a higher range of feedback for the patient, in addition to the proposed solution presented within this article.

The present work focused on implementing virtual reality technology by developing a tool to enhance physiotherapy practices, providing more effective exercise routines while addressing issues of monitorization and execution vigilance, without compromising the safety of patients or physicians. The aim of this project was to identify the tracking capabilities of the Oculus Quest system, particularly its potential for tracking patient’s chest to develop objective, data-driven monitoring for shoulder rehabilitation. This was achieved through the development of a virtual reality system designed to monitor exercises and provide real-time feedback on posture—one of the most critical factors for rehabilitation success.

## 2. Materials and Methods

### 2.1. Hardware Used

The materials used for the test were an Oculus Quest 2, Meta Platforms, Inc., Menlo Park, CA, USA. head-mounted display (HMD); two Oculus Touch controllers, whose characteristics are described in Table 1; and a tool developed to place one Oculus Quest controller in a way that allowed us to obtain the posture desired for the exercise.

A second system was used to track the position of the controller and headset, consisting of the Polaris Vega TX, Northern Digital Inc., Waterloo, ON, Canada, which is an optical tracker. It has a volumetric accuracy of up to 0.12 RMS, latency of less than three milliseconds, and frame rates as high as 400 Hz.

### 2.2. Software Used

The software used for this research was Unity for the development of the test and the creation of the system for posture analysis within Oculus Quest 2, as well as the APIs for the collection of data from the Polaris Vega.

The application developed for the Oculus Quest 2 was built within the VR Game default setup, taking advantage of the Oculus Integration within the Unity Assets store. In terms of resources, the software developed was a simple application in which the first scene consisted of a canvas menu and the option to access the two tests developed. Once the test is complete, a check mark indicates the particular test that has been completed.

Both tests were located in different scenes, but we used the same structure of data collection with scripts for every Oculus Quest execution cycle. We collected the data from the headset and the controllers for coordinates x, y, z, and the quaternions. In the case of the second test, additional data was collected relative to the interaction with the posture tracking. This data was then saved within the memory of the headset for later processing.

The software developed for the Polaris Vega collects the data from the Polaris system in the same terms and characteristics as the Oculus Quest and, afterwards, a timestamp is added for better synchronization of the data from both systems.

### 2.3. Tests Methodology

#### 2.3.1. Occlusion Test

The occlusion test was designed to evaluate how accurate the Oculus Quest 2 was on tracking the controller placed on the chest for position tracking and posture.

#### 2.3.2. Characterization of the Test

For the occlusion test (T1), a game was developed as a Unity application in which the participant introduced in the virtual reality environment should see a group of spherical shapes placed in the surrounding 3D space. These spheres were placed in a way to test the various headset positions according to the head movements of the patient relative to the controller placed on their chest. The participant uses their neck movements to reach each of the spheres with a headset pointer. The goal was to evaluate the accuracy of the Oculus Quest 2 to keep track of the controller placed on the chest.

**Figure 1:**
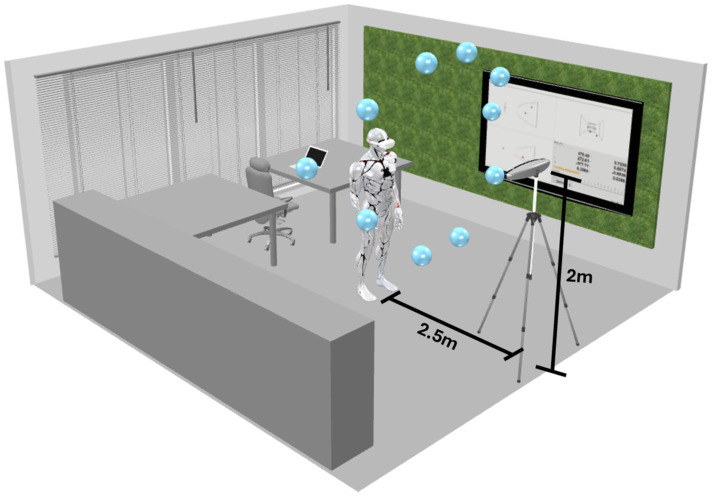
Experience representation: A visual depiction of the occlusion test. The ten blue spheres were evenly spaced along a circular arc around the headset in the virtual environment within the headset with a distance to the headset of approximately 2 m, inclined at 65 degrees both upward and downward from the front relative to the headset’s horizon line. The Polaris Vega was positioned directly in front of the headset at a distance of 2.5 m and a height of 2 m.

The cloud of spheres in the 3D environment was placed at a position that made the patient apply a neck rotational movement of around 65 degrees upward or downward to reach the different points, placing the headset in a certain position relative to the controller. Each position was performed for an interval of five seconds. Figure 1 illustrates the experience, merging the virtual reality spheres with the real environment. We collected the coordinates of the controller in relation to the headset from two main sources: the Oculus Quest 2 and the Polaris Vega.

Figure 1 shows the environment in which the test was conducted as well as the different points that were placed in the 3D virtual environment within the Oculus Quest application.

**Figure 2:**
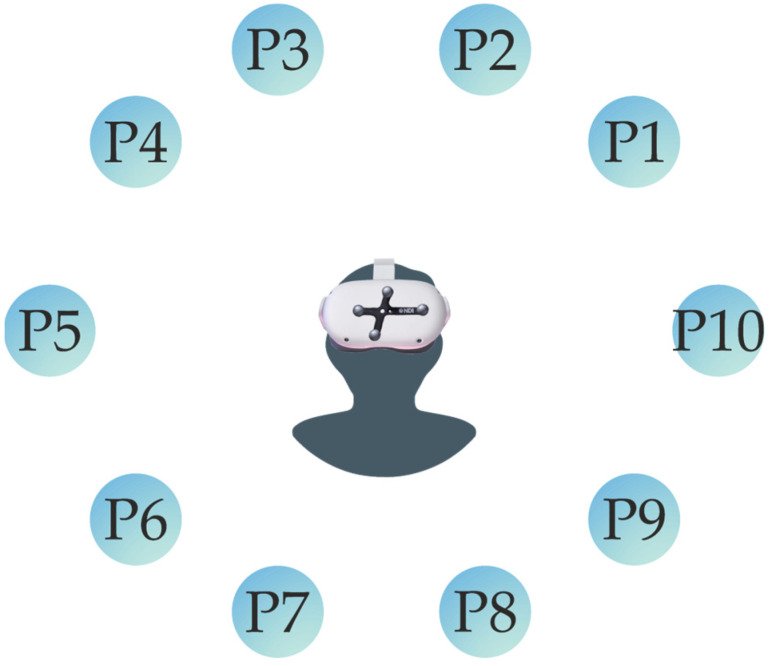
Visual representation of points sequence: Illustration of the test execution process, where the headset was sequentially aimed at each sphere from P1 to P10. The sequence followed the predefined arrangement of points, with the Polaris Vega’s origin reference tracker positioned on the headset.

Figure 2 shows that the sequence of the virtual spheres is fixed, and that the participant will pass through each point at a time in one specific order. Alongside the experiment, the Polaris Vega will record the information of the coordinates for both the controller and the headset. To collect this data, two passive tools were used: one was attached to the headset, as shown in Figure 2, and the other was a passive marker placed on the controller, positioned near the chest.

### 2.4. Exercise Test

**Figure 3:**
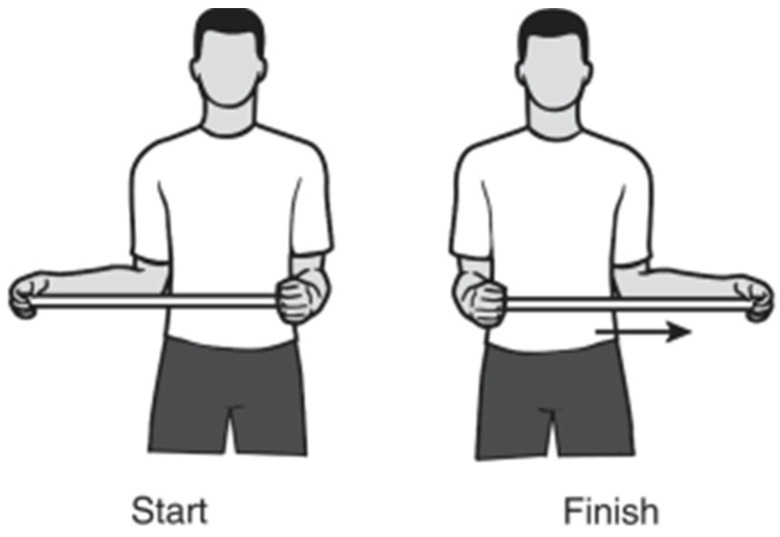
Passive external rotation exercise illustration adapted by the American Academy of Orthopedic Surgeons Rotator Cuff and Shoulder Conditioning Program [30].

To realize the exercise test, a previously validated physiotherapeutical exercise [30] for upper-limb rehabilitation was identified and selected (Figure 3). The goal was to execute an analysis of the performance of the position tracker solution, for which a series of sub-proceedings were introduced to characterize the impact of the solution.

The step-by-step directions of the exercise were established according to [30], as follows:

*“Step-by-step directions:*

- *Grasp the stick with one hand and cup the other end of the stick with the other hand.*
- *Keep the elbow of the shoulder you are stretching against the side of your body and push the stick horizontally as shown to the point of feeling a pull without pain.*
- *Hold for 30 s and then relax for 30 s.*
- *Repeat on the other side.”*

The directions provided are clear, nevertheless the execution of these exercises with a perfect technique needs practice, since they are a conjunction of instructions to perform simultaneously and require attention, which is not constant over long periods of time. With the usage of technology, we can translate this set of instructions into an interactive game to constantly reinforce the technique needs in a subtle way and make the patient perform accordingly.

**Figure 4:**
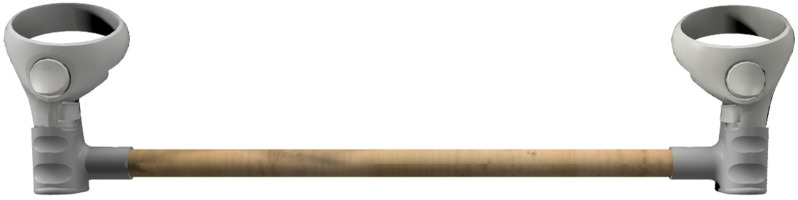
Exercise tool representation: Adaptation of the passive external rotation exercise using Oculus Quest controllers. The tool consisted of a 60 cm long wooden cylinder with a diameter of 2.2 cm. Two custom base adapters were attached at either end of the cylinder to securely hold the Oculus Quest controllers, enabling their integration into the exercise setup.

For this study, a tool was developed (Figure 4) to incorporate the Oculus Quest controllers into the exercise to monitor its execution.

The solution developed has several possibilities in terms of intervention to help the patient correct their posture or simply acknowledge their performance of the exercise. For this, the interventions were:

- Visual Feedback: The visual feedback consists of a 3D target that indicates where the body, or more specifically, the controller located on the chest, should be pointing towards.
- Haptic Feedback: The controller on the chest has vibration capabilities. This actuator will react according to the precision of the posture system relative to the target, allowing it to detect when a person is drifting from the ideal posture through the vibration on the chest.

Using the two solutions described, we introduced the following series of tests:

1. T2—Without haptic feedback.
2. T3—Haptic feedback without visual reinforcement.
3. T4—Haptic feedback full visual.
4. T5—Fast test without haptic feedback.
5. T6—Fast test with haptic feedback no visuals.
6. T7—Fast test visuals and haptic feedback.

The first three tests include a timer in each position of the exercise, so the participant needs to keep the controller in a still position for an interval of time before moving it to the next position. The reason why the last three tests are described as “fast tests” is because these three consist of a more fluid exercise execution, without timers.

**Figure 5:**
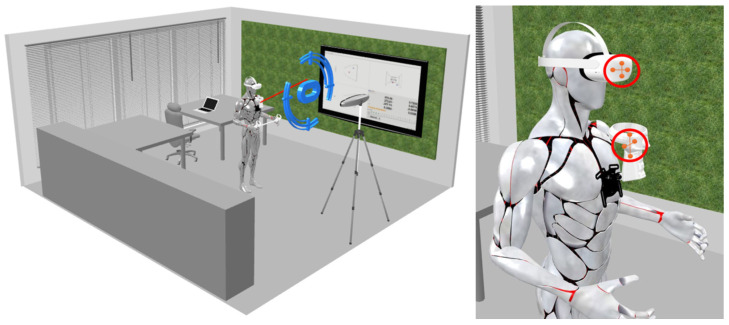
Experience representation: On the left, the blue target represents the virtual target used by the posture tracking system. On the right, the red circles indicate the placement of Polaris passive 4-marker rigid bodies utilized for tracking within the Polaris Vega, where the one placed on the headset works as the origin reference for the one placed on the chest controller.

Figure 5 illustrates the process used to analyze the performance during the execution of the exercise in the previously presented scenarios. This process utilizes the tool shown in Figure 4 and a controller positioned on the chest. The right part of Figure 5 highlights the placement of the Polaris passive markers.

When performing the exercises, we defined where the controller in the tool needed to be to ensure a specific position, and we ensured that the patient kept that position for a certain interval of time. The addition of these tools allowed us to define the precision with which the patient needed to keep the controller in the same position, providing extremely detailed feedback on the execution of the exercise.

### 2.5. Data Analysis

The data acquisition pipeline involved collecting data from two primary sources: the Oculus Quest 2 and the Polaris Vega. Data from the Polaris Vega was obtained in the form of a CSV file containing the coordinates of two passive trackers. For the Oculus Quest 2, the data was stored directly on the headset’s memory in a .txt file. The Oculus Quest software integrated both the tracking framework and the user interface to facilitate the execution of the tests including the occlusion and exercise execution tests. Regarding coordinate systems, both systems were configured to record coordinates in a similar manner, with the headset acting as the origin and using the passive markers on the headset and controllers to track their respective coordinates.

All data was analyzed using Python, specifically within Jupyter Notebook, utilizing libraries such as Matplotlib and NumPy for dataset management. Pandas was employed to ensure that the sampling rates of the two systems were aligned, as the Oculus Quest 2 has a higher sampling rate compared to the Polaris Vega. Both systems used timestamps that were previously synchronized to ensure proper data correlation.

The approach of this study involved calculating the standard deviation as a method of comparison between the two systems, aiming to analyze their repeatability and consistency. This analysis provided valuable and meaningful insights into the performance of each system. The standard deviation for both systems was calculated using Python scripts designed for data analysis. The calculation was performed using the following equation:

(1) $$\sigma =\sqrt{\frac{1}{N}\;\sum_{i=1}^{N}{\left(X_{i}-\mu \right)}^{2}}$$

The formula for standard deviation, as shown in Equation (1), involves determining the square root of the variance. The variance is calculated as the average of the squared differences between each data point ($X_{i}$) and the mean (*μ*), divided by the total number of data points (*N*). This method is crucial for quantifying the variability within each dataset and enabling a comprehensive comparison between the systems.

For the exercise posture tracking system, the interaction between the controller positioned on the chest and the target was recorded in two dimensions, similar to the movement of a pointer. Additionally, the Euclidean distance between the controller and the target was calculated, providing valuable and robust information about the execution of the exercise.

## 3. Results

The goal was to evaluate whether the solution developed can provide useful information regarding the position of patients throughout a series of experiments as well as to define the impact that the different components, both visual and haptic, have on the patient during the execution of the rehabilitation exercise.

For the tracking data collection, aside from the Oculus Quest 2, the second system to track was the NDI Polaris Vega^®^, a device able to perform optical tracking. In simple terms, this technology consists of a 3D visual tracking system that will allow us to have a reference for comparison with the Oculus Quest 2 tracking readings.

### 3.1. Occlusion Test Analysis

As previously stated in the last section, the occlusion test had the objective of detecting tracking issues within the Oculus Quest 2 controller placed on the chest of the patient (Figure 1).

**Figure 6:**
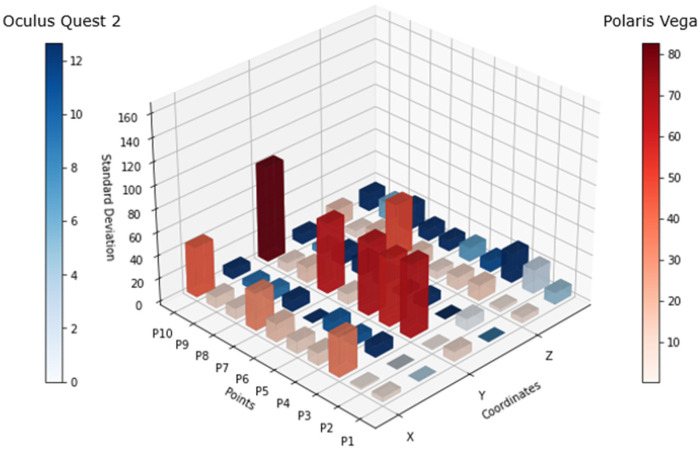
Standard deviation comparison: Comparison of the standard deviations for each point and coordinate across both systems during the occlusion test. The data is presented separately by axis, with red representing the Polaris Vega system and blue representing the Oculus Quest system.

Figure 6 shows the standard deviation of both tracking systems for each point, where the blue bars are relative to the Oculus Quest 2 tracking system and the red bars are from the Polaris Vega tracking system. For the scale comparison in both solutions, there was a big difference between these technologies, as there was a high variation in the Polaris system in comparison to the Oculus tracking coordinates.

The data was collected from a total of ten people with the goal of obtaining more data and performing a more robust analysis. The participants included in the study were selected by different body morphologies to validate a broader performance of the system.

**Figure 7:**
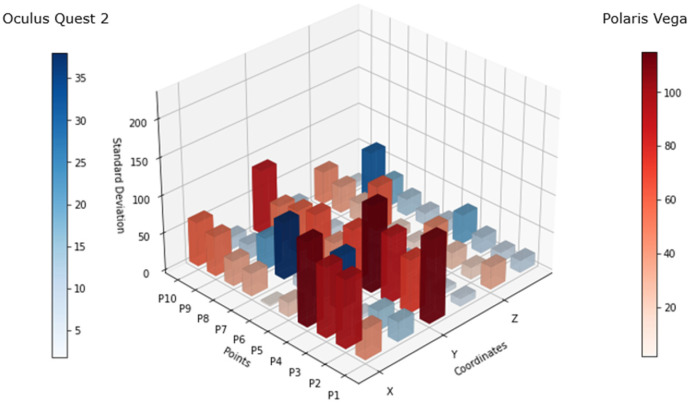
Standard deviation differential: A comparison of the standard deviation differentials derived from the data collected from 10 participants during the occlusion test. The results are color-coded, with blue representing the Oculus Quest system and red representing the Polaris Vega system.

Figure 7 shows the differential of the standard deviation (difference between the maximum and minimum standard deviation values) for each point, considering the values of all 10 subjects. The scales for both systems maintained a variance in resemblance to that in Figure 6, however, the behavior of the Oculus Quest 2 in terms of tracking precision showed an increase in standard deviation at some points, as can be seen at P4, P7, and P9. This lack of precision was visible in the x and z axes, more frequently in x axis.

### 3.2. Exercise Test Analysis

For the exercise test analysis, we collected data pertaining to the controller’s position coordinates. Additionally, we gathered the bidimensional coordinates of the interaction between the chest-placed controller and the posture target to analyze the steadiness of the patient throughout the entire execution of the exercise.

**Figure 8:**
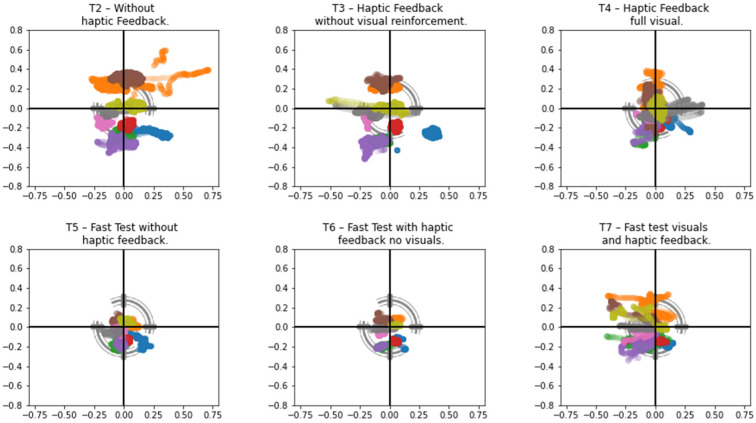
Posture analysis data from various tests conducted using the tracking framework. Each color corresponds to a specific test subject, illustrating variations in posture across exercises and individuals.

Figure 8 shows the behavior in terms of the movement of the trunk of the patient, where different colors represent different subjects. The differences in behavior could be seen throughout the different tests, for example, for the first three (T2, T3, and T4), the exercise was controlled by the application in terms of position control and timing, following the instructions previously quoted and not allowing the patient to advance freely until they completed the specified time interval in each position. The other three tests (fast tests: T5, T6, and T7) were performed without the timer, allowing the subject to perform the exercise without the need to maintain the position for a certain amount of time.

**Figure 9:**
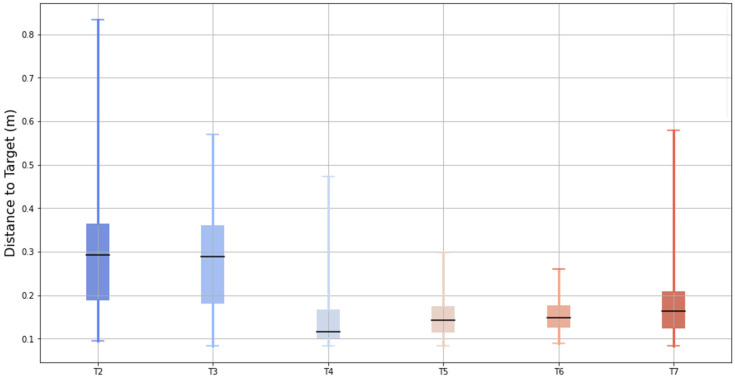
Bidimensional distance between the chest controller pointer and the target center across tests (T2–T7), showing the medians, interquartile ranges, and variability.

Figure 9 illustrates the bidimensional distance between the pointer created by the chest controller and the center of the target across six test conditions (T2 to T7). The box-and-whisker plot displays the median (central black bar), interquartile range (IQR, the box), and the data range excluding outliers (whiskers).

The results showed a progressive reduction in the median distance from T2 to T7, indicating improved posture alignment with the aid of the feedback tools. While T4 showed lower median distances compared to T5, T6, and T7, its variability (IQR and whiskers) was notably larger, suggesting less consistency in the posture during that test. These findings support the conclusion that the feedback tools contribute to improved and more consistent posture performance in later tests.

## 4. Discussion

The results of this study demonstrate the potential of virtual reality (VR) systems, like the Oculus Quest 2, as effective tools for enhancing shoulder rehabilitation exercises. By providing precise tracking of the controller and headset movements, the VR system successfully addressed challenges in posture monitoring and feedback delivery, even under occlusion or “blind spot” conditions. This suggests that VR-based solutions could overcome some of the key limitations of traditional physical therapy such as the lack of real-time feedback, limited precision in monitoring movement, and inconsistent adherence to proper technique.

The analysis of Figure 6 and Figure 7 highlights discrepancies in the tracking accuracy between the Oculus Quest 2 and the Polaris Vega system. While the Polaris system demonstrated some limitations in maintaining consistent tracking of passive markers, the Oculus Quest 2 sustained reasonable precision under various conditions. This ability to track movements even during occlusion or “blind spot” scenarios is crucial for physical therapy, where precise posture monitoring is essential for effective rehabilitation. Compared to traditional PT approaches that rely heavily on subjective observation or less interactive methods, this VR-based approach provides an objective, data-driven framework for assessing and correcting posture.

Figure 7 shows that there was constant variation in the tracking, which was significantly superior when the headset was facing down on both sides (left and right). This means that there might be some spots that have to be within the proximity of the controller to the headset, considered as “blind spots”, where the precision of the controller position might oscillate. This mostly occurred in the x axis and could also occur in the z axis, as the data showed.

The exercise study (T2–T7) demonstrated that participants progressively improved their performance with the help of VR feedback tools. Early tests, such as T2 and T3, showed significant variation in posture due to the lack of familiarity and technique. However, the introduction of real-time haptic and visual feedback (Figure 5) helped the participants make steady corrections toward the ideal posture in subsequent tests (T4–T7). This highlights the unique advantage of VR systems in providing immediate and actionable feedback, a feature that is often missing in traditional PT. By enabling participants to self-correct in real-time, the VR system fosters greater autonomy and accelerates the learning curve for proper technique.

Figure 8 shows the results for different persons performing the exact same exercise under the same conditions. With the obtained data, we could draw a first layer of conclusions on the comparison between tests, where the first test showed the highest level of variation, representing a lack of practice and technique that was then developed by performing the exercise repeatedly.

The instructions of the exercise were clear at the beginning of the experience, and as the person progressed throughout the series of tests, there was an improvement in the movement. T3 showed more steady movements as the haptic feedback provided indications to the participant, but haptic feedback on its own did not directly contribute to enhance or correct the posture, although it proved useful when identifying variations. In T4, we could see that while there was an attempt and visible correction toward the ideal posture, nevertheless, there was added movement.

Tests T5 to T7 showed progressed improvement toward maintaining posture. It is important to remember that these tests were conducted in sequence without any pause, so after applying visual feedback to the subject, they were able to sustain a more fixed posture without any feedback.

The study also explored the effect of time control on exercise execution. In tests without enforced timing, the participants tended to move more quickly and with less precision, mimicking the unsupervised exercise conditions often seen in traditional PT settings. The VR system, however, proved effective in mitigating this issue by providing structured visual and haptic feedback to guide movements, as seen in the improved performance during T5–T7. This underscores the potential of VR systems to provide consistent supervision and ensure adherence to proper techniques, even when the participants perform the exercises independently.

Figure 9 shows how the best performance in terms of mean distance to the target was achieved by applying a control not only in terms of the virtual reality scenario, but also by providing the full capacity of this posture tool.

The number of data points collected across the tests correlated with the duration of data collection. Faster tests, such as T4 and T6, resulted in fewer data points, which may have led to a mistaken interpretation of “better performance”, as shown in Figure 8. This highlights the importance of defining the best practices for exercise design within the virtual reality framework to ensure accurate performance evaluation.

Overall, the results of this study support the viability of VR systems as effective tools for enhancing physical therapy outcomes. By combining precise posture tracking, real-time feedback, and structured exercise protocols, the VR system addresses key limitations of traditional PT approaches including inconsistent feedback, the lack of objective tracking, and challenges with unsupervised adherence. Moreover, the ability of the VR system to adapt to different conditions, such as timed or untimed exercises, makes it a versatile solution for rehabilitation settings. Future research could further explore how integrating these systems into clinical practice might improve the long-term outcomes for shoulder rehabilitation patients.

## 5. Conclusions

The discussion presented in this article points towards the positive capacity of the usage of the Oculus Quest 2 as a tool for upper-limb rehabilitation. Throughout a series of tests, we validated the capacity and precision of the system for the extraction of relevant information and to induce a series of instructions on the person’s behavior to make them capable of following the instructions in a less tedious way. This was achieved by implementing dynamic solutions that could be transformed into a gamifying tool capable of maintaining the engagement of the patient in the realization of the exercises, without directly referring to the real goal of the task they are performing. This enabled them to abstract from the tedious and repetitive aspects of physiotherapy exercises.

This research analyzed the impact of a tool designed to correct and monitor the patients’ posture during exercises, demonstrating that the solution effectively enhances exercise performance and provides valuable insights. The tool offers a dynamic approach to patient engagement, ensuring the accurate execution of movements while also collecting meaningful data for physicians. This data can be analyzed to assess the patient’s progress and inform future therapeutic decisions.

## Figures and Tables

**Table 1:** Oculus Quest 2 device characteristics.

Specifications	Information
Memory	6 GB
Storage	256 GB
Display	LCD 1832 × 1920 per eye @ 72–120 Hz
Graphics	Adreno 650 (~1.2TFLOPS)
Sound	2 built-in speaker/3.5 mm headphone jack 6DOF. Inside-out tracking through 4 built-in cameras and 2 controllers with accelerometers and gyroscopes
Input	
Controller Input	Oculus Touch
Camera	4 infrared cameras
Mass	503 g

## Data Availability

The data supporting the findings of this study are available from the corresponding author upon reasonable request.
